# The Baneful Effects and Curability of Catarrhal Inflammations of the Upper Respiratory Tract*Read before the Georgia Medical Association at Augusta, April, 1901.

**Published:** 1901-06

**Authors:** Arthur G. Hobbs

**Affiliations:** Atlanta, Ga.


### THE BANEFUL EFFECTS AND CURABILITY OF CATARRHAL INFLAMMATIONS OF THE UPPER RESPIRATORY TRACT.*

By ARTHUR G. HOBBS, M.D.,

ATLANTA, GA.

By the phrase catarrhal inflammation of the respiratory tract we mean all those inflammatory conditions of a catarrhal nature which we find in the mucous membranes of the nose, nasopharynx, pharynx, larynx, trachea and the larger bronchi. Any inflammation of a mucous membrane, whether it be simple or complicated by local results, interferes with, at least, the functional activity, if it does not, as it usually does later, pervert its physiological function to a greater or less extent.

Inflammations of mucous membranes have a tendency to, and if allowed to remain unchecked, often do extend by continuity, either directly or through the baneful effects of contaminating excretions.

Auto-resolution is the rule in acute inflammations of mucous membranes when not repeatedly subjected to the original cause, but since the first cause repeats itself in the majority of cases, it

* Read before the Georgia Medical Association at Augusta, April, 1901.

may be expected that without assistance the acute stage will pass gradually into the subacute, then into the chronic and then in some localities into the atrophic, with the baneful sequences of the two latter especially.

The rules of mucous membrane pathology are applicable in a greater or less degree to all the mucous membranes of the body as well as to those of the upper respiratory tract.

That the nose was intended by nature only as an olfactory organ is the accepted belief of the laity. But, that so many of the profession should yet entertain the same idea is more to be wondered at. It would seem that many doctors have never taken the time to allow it to occur to themselves that olfaction is only a secondary function of the nose and that the space allotted to this least important of the senses is confined to a very small part of this most prominent feature. As nature's outlet of the respiratory tract any perversion of its functions naturally exert a baneful influence upon the deeper portions of this tract, whether this perversion be due to the inflammatory state or to its chronic results.

As an automatic organ, there are but few in the body to compare with the work performed by the lining membrane of a healthy and natural nasal mucous membrane. It supplies the proper moisture and warmth to adapt the air to the lungs by secreting an immense amount of fluid for the dry and cold air to pass over, or a much less amount when the air is moist and warm, thus automatically adapting itself to the humidity and temperature of the inspired air. As in the gills of a fish, nature has supplied a large superficial area of mucous membrane surface to this upper part of the respiratory tract—about twenty-four square inches.

Binz, Onodi and others have estimated that as much as twenty-four ounces of fluid are secreted daily by this large surface when the air is devoid of humidity in order to supply it with sufficient moisture to make it suitable to the parts below where the mucous membrane is less active in its secretory function, and hence less automatic in its response to the necessities of a uniform degree of humidity and warmth of the inspired air.

Nasal stenosis, as the result of some form of catarrhal inflammation of the mucous membrane of that organ and its contiguous parts, whether directly due to turbinal thickenings, accumulations, adenoid growths or tonsillar enlargements, necessitates mouth-breathing, at least during the sleeping hours, in adults, and continuously in children. In mouth-breathing the inhaled air cannot pass over more than half of the surface that it does when inhaled through the nose, and this small surface is insufficient to impart the proper moisture and temperature. Hence, how natural the dry throat and parched mouth of the little sufferer in his vain effort to get nutritious blood; and when we add to this the baneful effects of the contaminated air on the lining membrane of the bronchi and lung cells, it would not be a stretch of the imagination, should we reason by influence alone, without pausing to consider the many well-known and easily demonstrable results, how disastrous are the constant and continued inflammations of the upper respiratory tract not only to the lower air passages, but to the whole growing body. Moreover, the child's general nutrition is so impaired that his physical development is stunted and he becomes an easy prey to the zymotic and other diseases incident to childhood. What is true of the child is true in a measure to adulthood. The young adult has not only failed to fortify his breathing apparatus against hereditary tendencies, but he has actually rendered it a culture field for tubercle and the other bacilli. It is not necessary to follow up the pathological results of this never-ending battle of the upper respiratory mucous membrane for physiological supremacy. I might have said "of this organ," since it has such distinct and vital functions to perform.

Acute and chronic rhinitis in some of their different phases, usually in that of the recurrent, are the causes by extention or by pressure of nearly all eustachian tube and middle-ear diseases, the great majority of which results could be prevented, or at least checked, if properly treated in the beginning. Especially do I believe it true that 75 per cent. of the mastoid operations that are now performed would be unnecessary if the inflamed nasal and pharyngeal mucosa were properly treated from the beginning.

I have recently seen in a Berlin ear clinic seven mastoid operations performed on babies during the morning hours of one day. The operations were performed with a few whiffs of chloroform and a prune-shaped gouge. In most of the cases I saw nothing but bloody serum and blood. There had been apparently no attempt at any other form of treatment. The operator explained that it was a better form of counterirritant, anyway, than a blister. I have always realized the great efficacy of mastoid operations when distinctly indicated, but it had never occurred to me before that the operation should be resorted to as a counterirritant.

Catarrhal conjunctivitis is another of the evil consequences of the extension through the lachrymal duct, and it sometimes proves to be intractable until proper sprays are applied to the nasal end of the duct.

I am confident that lymphoid tissue growths in young scorbutic children can be arrested, if not aborted, before the adenoid vegetations have gained sufficient size to obstruct the channel. I am confident, also, that I have done this in a number of cases by the judicious use of vaseline sprays containing some mild astringent resolvent or antiseptic, together with alterative tonics. When, as a result of inflammation in the surrounding parts of the tract, adenoid vegetations assume a sufficient size to produce an obstruction, the now common operation of removal is indicated and is most effectual in its results.

I realize that I do not agree with most operators in not finding it necessary to extricate the whole of the mass, although I consider it desirable. I saw Dr. Dundas Grant, of London, last summer make eight adenoid operations in one hour on children. He told me he had made seventeen adenoids in one hour about a month before at one of his Friday clinics, exclusively for adenoids and tonsils. He used only a few whiffs of nitrous oxide on the little patient, and had them firmly held by assistants. This would suggest that he considered it necessary to remove every vestige of the growth.

With a careful use of cocain first, then the free use of orthoform, the operation is not a painful one, and I rarely use a general anesthetic even in the youngest child. I usually use my own fenestrated forceps first, then, if necessary, scrape with a malleable ring curette. A spray of suprarenal extract may or may not be necessary either before or after the operation. I find that what particles of the vegetation may be left can be made to slough or atrophy by the judicious use of the vaseline spray medicated properly.

Nasal deflections, spurs, turbinal hypertrophies, polypi, etc., by their mere obstructive presence cause mouth-breathing with all the baneful effects to the lower air passages that follow that most uncomfortable condition, together with these direct effects of mouth-breathing; we have these effects from the impure air inspired and from the degenerated secretions that flow from the parts contiguous to the obstruction.

Hence, all such obstructions should be speedily corrected for the good alone that would follow to the lower air passages, to say nothing of the great comfort that easy and natural breathing affords. About fifteen years ago the pendulum swing in the indiscriminate use of nasal saws, gouges and drills had about reached its height and then took a backward course, until about five years ago; the saw, in a more adaptable shape, began to regain its former place, but still with more discrimination, and particularly is this true in the resection operation of enlarged turbinate bones. But I think there has been no such reaction in the use of gouges and drills. In my opinion these are being laid aside for rare necessities. It is my observation that the galvanocautery has had about the same pendulum swing as the saw during the past fifteen years. I have used it on turbinals for eighteen years, but only by a knifelike cut through the mucous membrane to form an eschar in the tissue near the turbinal bone. This is the manner in which the operation is now generally performed instead of by the old destructive method to the mucous membrane.

Enlarged tonsils produce a train of disasters in the growing child that are not found alone in the thorax, but they retard development and weaken resistance to subsequent attack of acute diseases. I have amputated several thousand tonsils, and I do not recall a single case in which the result was not all that could be desired, neither have I ever had a case of alarming hemorrhage. I am aware that this is claiming much for amputation, when perhaps the majority of laryngologists believe in enucleation, whether they practice it or not.

When the American Rhinological Association was organized in

1879 by the efforts principally of Dr. Thos. F. Rumbold, the subject of nasal sprays formed quite naturally the chief topic of its papers and discussions for the first two or three years of its existence.

By a consensus of opinion of its members, vaseline was given the preference as a basis for sprays. Among other reasons because of its soothing and emollient effects upon inflamed mucous membranes. Its peculiar osmotic properties, as well as its miscibility with the essential oils and with many of the alkaloids, when skillfully used with properly selected ingredients suitable to each individual case, makes it a valuable adjunct in all perverted conditions of the mucosa, not only of the upper respiratory tract but of the bronchi also. It stimulates endosmosis in all columnar and columnar ciliated epithelium. But when an exosmotic effect is desirable, as in acute hyperplasias, a spray containing Glyco-Thymoline or some kindred antiseptic and resolvent is preferable. However, when it is desirable, and it often is so, to apply medicaments, such as beachwood, creosote, oil of terebene, etc., to the bronchial mucous membrane direct, it is more convenient to use some of the more fluid petroleum extracts as the accipient. All vaseline or oil sprays should be made with a fine-pointed atomizer, such as the DeVilbis, when used in the office, but when prescribed for the patient's use at home I think it preferable to prescribe my own, called the Greenwood atomizer, which is made of glass annealed to a toughness which resists easy breakage and can be made perfectly antiseptic by placing in boiling water.

Why should not direct medication be more frequently resorted to for the immediate effects upon the lining mucous membrane of the lower respiratory tract as well as to the upper? My experience is that most gratifying results do follow from such inhalations of finely atomized oily fluids properly medicated. Only an oily menstruum (vaseline, albolene, or some other of the petroleum products), is practicable for the parts below the larynx, since deliquescence takes place more readily with aqueous sprays, hence its greater tendency to produce strangling.

The use of this form of spray was taken up more slowly in the East perhaps from their idea that "no good could come out of

Nazareth," the West and South having been the prime movers in its introduction. But I have recently found that the so-called vaseline sprays, which includes all that have for their basis any of the oily products of petroleum, are now being used quite extensively abroad, and in London and Paris particularly did I find that the inhalation form of these sprays was also resorted to in the treatment of irritations and obstructions of the bronchial tubes.

I have said this much on the subject of the spray because I regard it as one of our best instruments not only in curing inflammatory conditions of the upper respiratory tract, but in the treatment of the baneful results therefrom.

